# Novel *STAR* gene variant in a patient with classic lipoid congenital adrenal hyperplasia and combined pituitary hormone deficiency

**DOI:** 10.1038/s41439-021-00138-w

**Published:** 2021-02-03

**Authors:** Moritake Higa, Akiko Zaha, Akiko Takushi, Nami Morishima, Toyofumi Majikina, Takeshi Touma, Michio Shimabukuro, Hiroaki Masuzaki, Misa Honda, Tomonobu Hasegawa

**Affiliations:** 1 Diabetes and Lifestyle-Related Disease Center, Tomishiro Central Hospital, Okinawa, Japan; 2 Department of Diabetes, Endocrinology, and Metabolism, Fukushima Medical University, Fukushima, Japan; 3 Division of Endocrinology, Diabetes and Metabolism, Hematology, Rheumatology, Department of Medicine, Graduate School of Medicine, University of the Ryukyus, Okinawa, Japan; 4 Department of Pediatrics, Keio University School of Medicine, Tokyo, Japan

**Keywords:** Genetics research, Adrenal gland diseases

## Abstract

We report the first case of classic lipoid congenital adrenal hyperplasia and combined pituitary hormone deficiency. We identified pathogenic variants in the *STAR* gene: a novel variant of c.126_127delCCinsG, namely, p.Thr44Profs*2 and an already reported variant of c.634C>T, namely, p.Gln212*. The association with combined pituitary hormone deficiency might be just a coincidence.

Lipoid congenital adrenal hyperplasia (LCAH; OMIM 201710) is an autosomal recessive disease caused by biallelic pathogenic variants in the *STAR* gene^1^. STAR is expressed in steroidogenic cells in the adrenals and gonads, facilitating transport of cholesterol from the outer to inner mitochondrial membrane, where the first step in steroidogenesis commences. LCAH is endocrinologically characterized by impairment of all classes of steroidogenesis in adrenals and gonads. LCAH is composed of the classic type and nonclassic type^2^. Patients with classic LCAH develop early infantile-onset primary adrenal insufficiency. 46,XY patients show complete female external genitalia, and 46,XX patients achieve thelarche and menarche spontaneously, although menstrual cycles are often anovulatory and menopause occurs early. Patients with nonclassic LCAH develop relatively late-onset primary adrenal insufficiency without any episode of salt loss. 46,XY patients showed complete male external genitalia, and 46,XX patients can achieve spontaneous pregnancy and delivery^3,4^.

Combined pituitary hormone deficiency (CPHD) is a congenital condition that causes deficiencies in more than two hormones produced by the pituitary gland. Approximately 30 genes have been identified as responsible for CPHD^5^. We report, for the first time, a Japanese female with classic LCAH and CPHD. We identified a novel variant of c.126_127delCCinsG, namely, p.Thr44Profs*2 and an already reported mutant of c.634C>T, p.Gln212* in the *STAR* gene.

A Japanese female had frequent vomiting 1 week after birth and generalized seizures at the age of 3 months. Reportedly, she was clinically diagnosed with primary adrenal insufficiency, although her medical record was discarded. Glucocorticoid and mineralocorticoid supplementation therapy was started. At 2 years of age, she had generalized skin pigmentation. Her karyotype was 46,XX. Her endocrinological data are summarized in Table 1 and were compatible with primary adrenal insufficiency. At the age of 12 years, she presented with menarche, with no subsequent menstruation. At the first visit to our hospital at the age of 35 years, her height was 147 cm (−2.0 SD), and her body weight was 95 kg (+5.4 SD). She had taken daily doses of hydrocortisone 15 mg, fludrocortisone 0.1 mg, and dexamethasone 0.5 mg. On examination, her blood pressure was 135/85 mmHg. Her breast and pubic hair were Tanner I. The intelligence quotient score was 45. She had right low-frequency sensorineural hearing loss between 20 and 40 dB. Abdominal plain CT revealed a slightly atrophic uterus and seemingly normal ovaries. Pituitary magnetic resonance imaging revealed that her pituitary was slightly smaller than that of a female of reproductive age. The endocrinological baseline data are presented in Table 1. The gonadotropin-releasing hormone stimulation test showed that the basal/peak LH and FSH values were 3.61/9.20 mIU/mL and 7.76/11.20 mIU/mL, respectively. The growth hormone-releasing peptide-2 stimulation test and arginine stimulation test showed peak growth hormone (GH) values of 3.31 and 0.12 ng/mL, respectively. These data indicated that she had CPHD, namely, hypogonadotropic hypogonadism (HH) and adult GH deficiency (GHD).

**Table 1:** Endocrinological data.

	2 years of age	35 years of age	Adult reference range
Blood			
ACTH (pg/mL)	>800	20	<50
F (microg/dL)	<1	<1	3.8–13
DHEAS (microg/dL)	N.D.	20	18–391
PRA (ng/mL/h)	>25	11	0.3–2.9
PAC (pg/mL)	<10	<10	10.09–62.7 at supine position
Estradiol (pg/mL)	N.D.	21.92	28.8–196.8 at follicular phase
TSH (microIU/mL)	N.D.	1.96	0.350–4.94
FreeT4 (ng/dL)	N.D.	0.95	0.70–1.48
FreeT3 (pg/mL)	N.D.	2.81	1.71–3.71
PRL (ng/mL)	N.D.	14.32	1.3–25.0
IGF-1 (ng/mL)	N.D.	29	98–245
Urine			
17-KS (mg/day)	N.D.	1.2	2.4–11
17-OHCS (mg/day)	N.D.	2.0	2.2–7.3
Pregnanediol (mg/day)	0.05	N.D.	>0.28 at follicular phase
Pregnanetriol (mg/day)	0.01	N.D.	>0.13 at follicular phase

*ACTH* adrenocorticotropic hormone, *F* cortisol, *PRA* plasma renin activity, *PAC* plasma aldosterone concentration, *17-KS* 17-ketosteroids, *17-OHCS* 17-hydroxycorticosteroids, *DHEAS* dehydroepiandrosterone sulfate, *N.D.* not done.

After obtaining informed consent from the patient and her sister and with the approval of the Institutional Review Board of Keio University School of Medicine, genomic DNA was extracted from leukocytes of the patient. We PCR-amplified and sequenced the *STAR* gene. The primer sequences and PCR conditions were provided previously^6^. We identified a novel variant of c.126_127delCCinsG, namely, p.Thr44Profs*2, and an already reported mutant of c.634C>T, p.Gln212* (ref. ^7^) (Fig. 1). c.126_127delCCinsG was absent from databases, including the Genome Aggregation Database (gnomAD; https://gnomad.broadinstitute.org/), Human Genetic Variation Database (HGVD; http://www.hgvd.genome.med.kyoto-u.ac.jp/), Human Gene Mutation Database (HGMD; http://www.hgmd.cf.ac.uk/ac/index.php), and ClinVar (https://www.ncbi.nlm.nih.gov/clinvar/). Parental genetic analysis was refused. We also analyzed major or possible causative genes for CPHD and/or HH, namely, *AXL, CCDC141, CHD7, DMXL2, DUSP6, FEZF1, FGF8, FGF17, FGFR1, FLRT3, FSHB, GH1, GHR, GHRH, GHRHR, GHSR, GLI2, GNRH1, GNRHR, GPR161, HESX1, HS6ST1, IGF1, IGF1R, IL17RD, KAL1, KISS1, KISS1R, LHB, LHX3, LHX4, LEP, LEPR, MKRN3, NELF, OTUD4, OTX2, PAX6, PCSK1, PGM1, PNPLA6, POLR3A, POLR3B, POU1F1, PROK2, PROKR2, PROP1, RNF216, SEMA3A, SEMA7A, SIX3, SIX6, SOX2, SOX10, SPRY4, STAT5B, STUB1, TAC3, TACR3, TBX19*, and *TUBB3*, using next-generation sequencing and the MiSeq instrument (Illumina, San Diego, CA, USA), according to the SureSelect protocol (Agilent Technologies, Santa Clara, CA, USA)^8^. No pathogenic variant among the tested genes was identified.

**Fig. 1:**
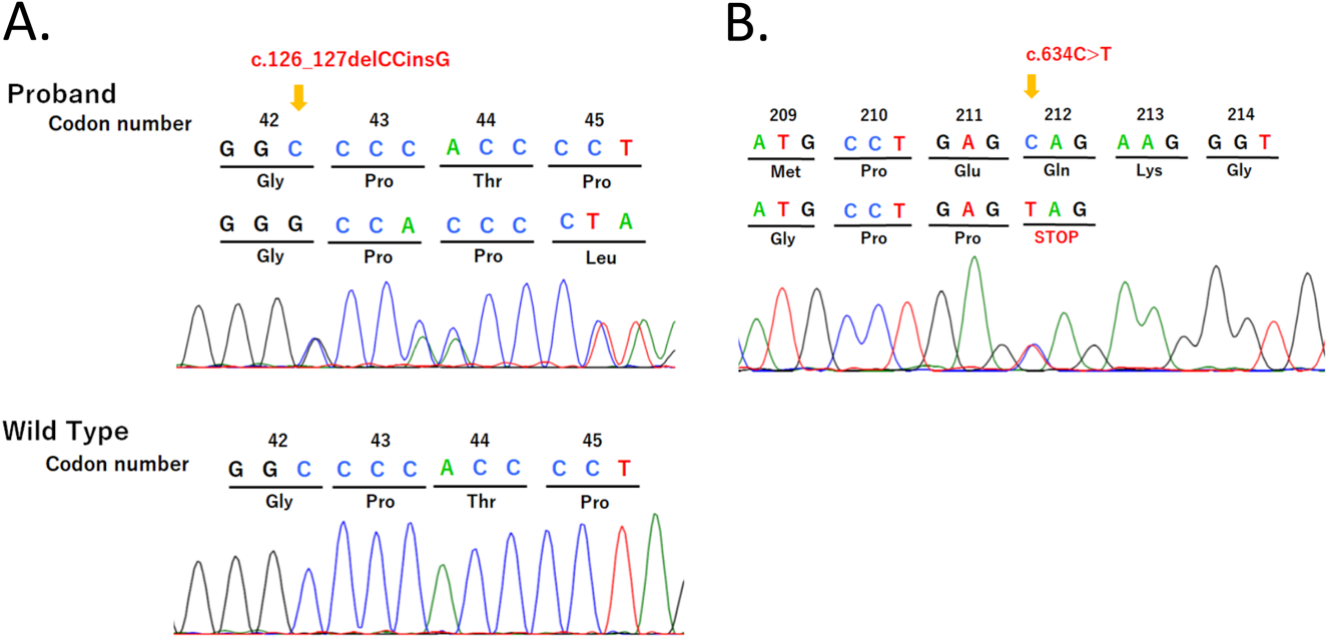
Partial chromatogram of the *STAR* gene. The left panel shows c.126_127delCCinsG in the proband as well as the wild type (**A**). The right panel shows c.634C>T in the proband (**B**).

The diagnosis of classic LCAH in this patient is convincing due to the clinical history of genetic variants in the STAR gene. Notably, one of them was novel: c.126_127delCCinsG. This variant must be pathogenic because it was not reported in the variant database and caused a stop codon, theoretically leading to nonsense-mediated mRNA decay in vivo. As parental genetic analysis was refused, we cannot prove compound heterozygosity.

She had an atypical clinical course, manifesting as LCAH. First, she had CPHD composed of HH and adult GHD. To the best of our knowledge, no case of LCAH and CPHD has been reported^4,5^. CPHD cannot be explained by *STAR* gene variants, as the STAR protein is not expressed in the pituitary gland (https://www.proteinatlas.org/ENSG00000147465-STAR/tissue). The onset of CPHD may be around pubertal age, judging from her height (147 cm) without any growth-promoting treatment and the presence of menarche. At present, the cause of CPHD is unknown, as no variant was identified in major or possible causative genes for CPHD and/or HH. Potential causes of CPHD include a monogenic disorder, which has not been established as a disease entity, secondary sequelae due to hypoglycemia by early-onset adrenal insufficiency, and/or others. The association between classic LCAH and CPHD might be just a coincidence. Second, she had mental delay. This might be secondary sequelae due to hypoglycemia. Third, she had right low-frequency sensorineural hearing loss, again, probably due to secondary sequelae due to hypoglycemia.

In conclusion, we identified a novel *STAR* variation in a patient with classic LCAH and CPHD.

## Data Availability

The relevant data from this Data Report are hosted at the Human Genome Variation Database at: 10.6084/m9.figshare.hgv.2963, 10.6084/m9.figshare.hgv.2966.
